# Exposure to Bullying and General Psychopathology: A Prospective, Longitudinal Study

**DOI:** 10.1007/s10802-020-00760-2

**Published:** 2021-01-22

**Authors:** Jolien Rijlaarsdam, Charlotte A. M. Cecil, J. Marieke Buil, Pol A. C. van Lier, Edward D. Barker

**Affiliations:** 1 Department of Child and Adolescent Psychiatry/ Psychology, Erasmus MC-University Medical Center Rotterdam, P.O. Box 2040, 3000 CA Rotterdam, the Netherlands; 2 Department of Epidemiology, Erasmus MC-University Medical Center Rotterdam, Rotterdam, the Netherlands; 3 Department of Clinical, Neuro and Developmental Psychology, Amsterdam Public Health Research Institute, Vrije Universiteit Amsterdam, Amsterdam, the Netherlands; 4 Department of Psychology, Institute of Psychiatry, Psychology and Neuroscience, King’s College London, London, UK

**Keywords:** Bullying exposure, General psychopathology, Longitudinal, ALSPAC

## Abstract

Although there is mounting evidence that the experience of being bullied associates with both internalizing and externalizing symptoms, it is not known yet whether the identified associations are specific to these symptoms, or shared between them. The primary focus of this study is to assess the prospective associations of bullying exposure with both general and specific (i.e., internalizing, externalizing) factors of psychopathology. This study included data from 6,210 children participating in the Avon Longitudinal Study of Parents and Children (ALSPAC). Child bullying was measured by self-report at ages 8 and 10 years. Child psychopathology symptoms were assessed by parent-interview, using the Development and Well-being Assessment (DAWBA) at ages 7 and 13 years. Bullying exposure significantly associated with the general psychopathology factor in early adolescence. In particular, chronically victimized youth exposed to multiple forms of bullying (i.e., both overt and relational) showed higher levels of general psychopathology. Bullying exposure also associated with both internalizing and externalizing factors from the correlated-factors model. However, the effect estimates for these factors decreased considerably in size and dropped to insignificant for the internalizing factor after extracting the shared variance that belongs to the general factor of psychopathology. Using an integrative longitudinal model, we found that higher levels of general psychopathology at age 7 also associated with bullying exposure at age 8 which, in turn, associated with general psychopathology at age 13 through its two-year continuity. Findings suggest that exposure to bullying is a risk factor for a more general vulnerability to psychopathology.

## Introduction

Bullying is a form of aggressive behavior that is repeated over time against one or more individuals who are relatively powerless (Monks et al. 2009; Salmivalli 2010). The aggressive behavior can take many forms such as name calling, hitting, spreading rumors, and social exclusion. Bullying is considered a significant public health problem world-wide, with prevalence rates ranging from approximately 10 to 25% (Analitis et al. 2009; Nansel et al. 2001; Thomas et al. 2017). There is mounting evidence that the experience of being bullied associates with overall mental health problems, including both internalizing and externalizing symptoms (Klomek et al. 2015; Moore et al. 2017; Reijntjes et al. 2010; Schoeler et al. 2018; Singham et al. 2017; van Lier et al. 2012).

However, it is unclear at present whether these identified associations with exposure to bullying are specific to particular psychiatric outcomes, or shared between them. Psychiatric disorders and their symptoms co-occur substantially, even across the broadly defined internalizing and externalizing domains (Achenbach et al. 2016; Angold et al. 1999; Kessler et al. 2005; Krueger 1999). Additionally, psychiatric disorders often have a multifactorial etiology that includes (i) shared risk factors, such as bullying, childhood maltreatment, maternal depression, and stressful life events (Caron and Rutter 1991; Kessler et al. 1997; Kim et al. 2003; Vachon et al. 2015), and (ii) a shared genetic vulnerability associated with, for example, the risk of exposure to bullying (Schoeler et al. 2019). In recent years, studies have suggested that this general vulnerability to psychopathology may be usefully represented by a bifactor model that captures (i) shared variance among a broad range of mental health problems (i.e., general factor), as well as (ii) specific influences beyond those explained by shared variance (i.e., specific factors) (for studies in children and early adolescents, see Lahey et al. 2015; Neumann et al. 2016; Olino et al. 2014; Patalay et al. 2015). Although in children the focus has often been on internalizing and externalizing dimensions of psychopathology, thought disorders (e.g., obsessive–compulsive disorders, schizophrenia) have been identified as a distinct third dimension in adolescents and adults (Caspi et al. 2014).

The general factor of psychopathology in childhood has been found to predict various psychiatric outcomes in adolescence, such as anxiety disorders, mood disorders, and substance abuse (Pettersson et al. 2018). The specific factors, however, predicted only a subset of psychiatric outcomes. For example, the specific internalizing factor associated with anxiety and mood disorders but not with, for example, substance abuse. Furthermore, previous research found evidence for both genetic and environmental influences on the general factor of psychopathology (Brikell et al. 2020; Brodbeck et al. 2018; Caspi et al. 2014; Neumann et al. 2016; Riglin et al. 2019). Of particular interest is the finding that childhood maltreatment (e.g., harsh discipline, physical abuse, sexual abuse) associated most strongly to a general factor of psychopathology as opposed to symptom specific factors (e.g., internalizing, externalizing) (Brodbeck et al. 2018; Caspi et al. 2014). However, to the best of our knowledge, no study to date has examined the association of bullying exposure in childhood with the general factor of psychopathology in early adolescence.

The primary focus of the current study is to assess the prospective associations of bullying victimization (8, 10 years) with both general and specific psychopathology factors (7, 13 years) while accounting for other risk exposures known to associate with overall mental health problems (e.g., childhood maltreatment, maternal depression, stressful life events). To this end, the aim of the study was threefold; (i) testing general psychopathology bifactor models at different time points across development, specifying a general factor in addition to specific internalizing and externalizing factors, (ii) systematically characterizing the chronicity and type (overt/relational) of bullying victimization in relation to both general and specific psychopathology factors, and (iii) incorporating the repeated assessment of bullying exposure and general psychopathology into a path model, together with the other risk exposures, to test for prospective interrelations.

## Methods

### Participants

Participants were drawn from the Avon Longitudinal Study of Parents and Children (ALSPAC). ALSPAC is an ongoing epidemiological study of children born from 14,541 pregnant women residing in Avon, United Kingdom, with an expected delivery date between April 1991 and December 1992 (85% of eligible population; Fraser et al. 2013). When the oldest children were approximately 7 years of age, an attempt was made to bolster the initial sample with eligible cases who had not joined the study originally, resulting in an additional 713 children being enrolled. Ethical approval for the study was obtained from the ALSPAC Ethics and Law Committee as well as Local Research Ethics Committees. Informed consent was obtained from all ALSPAC participants. The original ALSPAC sample is representative of the general population (Boyd et al. 2013). Please note that the study website contains details of all the data that is available through a fully searchable data dictionary: http://www.bris.ac.uk/alspac/researchers/data-access/data-dictionary/.

### Measures

#### Bullying Exposure

Exposure to bullying victimization was assessed at ages 8 and 10 years via child report, using the previously validated Bullying and Friendship Interview Schedule (Hamburger et al. 2011; Wolke et al. 2000). Trained psychology graduates asked children to rate victimization by peers during the past six months, using five stem/contingent question pairs on overt victimization (e.g., “having belongings stolen”) and four stem/contingent question pairs on relational victimization (e.g., “other children not wanting to play with them”). If children answered yes versus no (score 0) to any form of victimization, they were contingently asked how frequently it had occurred: 1 to 3 times in the past 6 months (infrequently, score 1), ≥ 4 times in the past 6 months but less than once per week (frequently, score 2), at least once per week (very frequently, score 3). The Bullying and Friendship Interview Schedule has demonstrated high inter-rater reliability and predictive validity (Zwierzynska et al. 2013). Using confirmatory factor analysis, the four relational victimization items showed acceptable internal reliability at age 8 (RMSEA = 0.020, CFI = 0.999, SRMR = 0.011) and age 10 (RMSEA = 0.010, CFI = 1.000, SRMR = 0.008). Similarly, the five overt victimization items showed acceptable internal reliability in a confirmatory factor analysis at age 8 (RMSEA = 0.026, CFI = 0.995, SRMR = 0.020) and age 10 (RMSEA = 0.025, CFI = 0.995, SRMR = 0.022). In line with previous research (Wolke et al. 2012), we computed continuous severity indices of exposure to bullying (overt and/or relational) by totaling the stem/contingent item pairs (each scaled 0–3) at 8 and 10 years, as well as across the two time points.

In follow-up analyses, we also examined categorical variables characterizing the chronicity and type (overt/relational) of bullying, based on previous research (Wolke et al. 2014; Wolke et al. 2012). Overt and relational bullying victimization was coded as present if children experienced victimization frequently or very frequently (Wolke et al. 2012). The following three categorical variables were constructed (Wolke et al. 2014, 2012): (1) *Any bullying victimization* (overt and/or relational) at 8 and/or 10 years of age; (2) *Chronicity of victimization*: never victimized (no report of victimization), unstable (reported at one time point), or stable (reported at both time points); (3) * Type of victimization* at 10 years: never victimized, victim of overt bullying only, victim of relational bullying only, victim of both relational and overt bullying. All categorical variables were dummy coded prior to analyses.

#### General Psychopathology

Psychopathology symptoms were repeatedly assessed with the Development and Well-being Assessment (DAWBA; R. Goodman et al. 2000), a validated semi structured interview. Parents completed open and closed questions about a range of symptoms relevant to youth psychiatric disorders, including both internalizing (generalized anxiety disorder [GAD], major depressive disorder [MDD], social phobia, separation anxiety, specific phobia) and externalizing (attention deficit hyperactivity disorder [ADHD], oppositional defiant disorder [ODD], conduct disorder [CD]) domains. For each disorder, an ordered categorical measure was generated using computer algorithms (A. Goodman et al. 2011), comprising six categories indicating the likelihood of each youth having the disorder from level 0 up to level 5. Using these DAWBA measures at ages 7, 10, and 13 years, we tested general psychopathology bifactor models specifying a general factor in addition to domain-specific internalizing and externalizing factors. See statistical analyses section for further details.

##### Covariates

All analyses adjusted for sex. We additionally accounted for a range of risk factors that may influence general psychopathology or have been routinely adjusted for in birth cohorts (Dunn et al. 2020). Specifically, we included previously established cumulative risk scores based on maternal reports (Cecil et al. 2014). For three developmental periods (i.e., pregnancy, early-childhood, late-childhood), risks were summed to create cumulative scores covering the following domains: (a) life events (e.g. death in family, accident, illness), (b) contextual risks (e.g. poor housing conditions, financial problems), (c) parental risks (e.g. parental psychopathology, criminal involvement and substance use), (d) interpersonal risks (e.g. intimate partner violence, family conflict), and (e) child maltreatment (e.g. child physically hurt or sexually abused, parent physically or emotionally cruel to child; available postnatally). As can be seen in Supplementary Tables S1.1-S1.3, the existing scores were slightly adapted to our study context, in such a way that (i) the bullying items were excluded, and (ii) the items were organized into one of three developmental periods so as to coincide with the general psychopathology data: pregnancy, birth–age 7 (early-childhood), and age 8–12 (late-childhood). The overall cumulative risk scores were estimated using confirmatory factor analysis, as described elsewhere (Cecil et al. 2014). In sensitivity analyses, we additionally corrected for child IQ as assessed at 8 years of age using the Wechsler Intelligence Scale for Children (3^rd^ UK edition).

##### Attrition

For this study, we included youth from ALSPAC who had available data on general psychopathology at age 13 years (*N* = 6,210, 49% male; see Table S2 for numbers, descriptive statistics, and correlations). In a multivariate model, we examined the extent to which the study variables (i.e., severity index of bullying exposure at ages 8–10, general psychopathology at age 7) and covariates (i.e., early-childhood risk exposure, IQ) associated with exclusion from the current study. Children with a lower IQ were more likely to be excluded in the current analysis (OR = 0.990, 95% CI = 0.986–0.994). However, bullying exposure (OR = 0.994, 95% CI = 0.982–1,006), general psychopathology (OR = 1,110, 95% CI = 0.999–1.234) and risk exposure (OR = 0.937, 95% CI = 0.868–1.012) were unrelated to exclusion from the study.

##### Statistical Analysis

The analyses proceeded in three main steps. In the first step, we used confirmatory factor analysis (CFA) to fit a general psychopathology bifactor model (Gibbons and Hedeker 1992), with the 13-year time point as the main time point of interest, measured prospectively with bullying exposure at ages 8 and 10. All analyses were repeated for the 7- and 10-year time points. In the bifactor model, each psychopathology subdomain loaded on a single general factor and on one domain-specific internalizing (GAD, MDD, social phobia, separation anxiety, specific phobia) or externalizing (ADHD, ODD, CD) factor. Because these general and domain-specific factors are modelled to be orthogonal to each other (Gibbons and Hedeker 1992; Rodriguez et al. 2016b), we constrained all factor covariances to zero. A general psychopathology bifactor model was computed in R version 3.4.3, using the package Lavaan (Rosseel 2012) with robust weighted least square mean and variance adjusted (WLSMV) estimation for ordinal data. We tested the replicability of this bifactor model in two ways. First, bifactor-specific indices (Rodriguez et al. 2016a; Rodriguez et al. 2016b) were computed using Marley Watkins’s “Omega” software, including: reliability estimates for both the general and specific latent factors (omega values), the proportion of variability in the latent factors that is explained by its indicators (H), as well as explained common variance (ECV) and the percentage of uncontaminated correlations (PUC) that offer information about the extent to which the data are multidimensional. Second, we directly examined the replicability of the bifactor structure in our longitudinal data (7, 10, 13 years). To inform the role of general psychopathology in our data, we repeated our regression analyses for a correlated two-factors model in which internalizing and externalizing factors each are indicated by a subset of psychopathology domains and are assumed to be correlated, but no general factor is identified (see Figure S1).

In the second step, we output factor scores from the bifactor model at age 13, saved them, and examined their associations with the continuous severity index of exposure to bullying across time points (age 8,10). In follow-up analyses, we also individually assessed categorical variables characterizing the chronicity and type (overt, relational, combined) of being exposed to bullying in relation to general and specific factors of psychopathology at age 13. The maximum likelihood estimation with robust standard errors (MLR) was used to correct for possible non-normal distributions of study variables.

In the final step, the continuous severity indices of exposure to bullying (ages 8, 10) and the saved general psychopathology factor scores (ages 7, 13) were then incorporated into a path model, together with the cumulative risk scores (pregnancy, early-childhood, late-childhood) to test for prospective interrelations. We used bootstrapping with bias-corrected 95% confidence intervals (10,000 bootstraps) to derive variance from the empirical distribution of the observed data. Model fit was established using the root-mean-square error of approximation (RMSEA: acceptable fit ≤ 0.08), the comparative fit index (CFI; acceptable fit ≥ 0.90), and the standardized root mean square residual (SRMR; acceptable fit ≤ 0.08) (Hu and Bentler 1999; Perry et al. 2015).

All analyses in steps 2 and 3 were performed in Mplus version 7.11 (Muthén and Muthén 1998–2016) using maximum likelihood estimation. We addressed missing data in our sample through multiple imputation (step 2) and a full information maximum likelihood (FIML) approach (step 3). In the individual models regressing general psychopathology on bullying exposure (step 2), missing data on covariates were imputed 20 times using the ‘data imputation’ procedure implemented in Mplus. The samples after multiple imputation ranged from *N* = 4,613 to *N* = 5,370 (see Table 1), depending on the specific bullying exposure variables being assessed. In sensitivity analyses, we compared associations for the continuous severity index of bullying exposure in the imputed (*N* = 5,370) and non-imputed (*N* = 5,125) data sets.

**Table 1:** Associations between exposure to bullying and general or specific factors of psychopathology

	Model A			Model B		
	GPF	INT	EXT	GPF	INT	EXT
Bullying exposure	β (SE)	β (SE)	β (SE)	β (SE)	β (SE)	β (SE)
Severity index (*n* = 5,370)^a^, score	0.12 (0.01)***	0.02 (0.01)	0.10 (0.01)***	0.07 (0.01)***	0.03 (0.01)*	0.04 (0.01)**
Any exposure (*n* = 4,613)						
yes (53.4%) vs no	0.12 (0.01)***	0.02 (0.01)	0.09 (0.01)***	0.06 (0.01)***	0.02 (0.01)	0.04 (0.01)**
Chronicity (*n* = 4,613)						
none (reference)						
unstable (41.0%)	0.10 (0.02)***	0.03 (0.02)	0.07 (0.02)***	0.05 (0.01)**	0.03 (0.01)*	0.03 (0.01)*
stable (12.4%)	0.12 (0.02)***	0.00 (0.02)	0.10 (0.02)***	0.07 (0.01)***	0.00 (0.02)	0.06 (0.02)***
Type (*n* = 4,882)^b^						
none (reference)						
overt only (16.1%)	0.09 (0.01)***	0.00 (0.01)	0.07 (0.02)***	0.06 (0.01)***	0.01 (0.01)	0.04 (0.01)**
relational only (2.8%)	0.04 (0.02)**	0.02 (0.02)	0.03 (0.01)	0.03 (0.01)	0.01 (0.01)	0.02 (0.01)
both (4.9%)	0.08 (0.01)***	0.01 (0.01)	0.06 (0.02)***	0.06 (0.01)***	0.02 (0.01)	0.04 (0.01)*

Model A presents linear regression results controlling for sex and early-childhood cumulative risk exposure; Model B is similar to model A, additionally controlling for pre-existing psychopathology (GPF, INT, or EXT)

GPF = general psychopathology factor; INT = specific internalizing factor; EXT = specific externalizing factor

*p < 0.05; **p < 0.01; ***p < 0.001

^a^Summed across time points (ages 8 and 10 years)

^b^Measured at age 10

Our integrative path model interrelating all study variables and covariates (step 3) offered maximum likelihood alternatives, such as the FIML approach. This FIML approach estimates a likelihood function for each individual based on the variables included in the model, and produces unbiased parameter estimates and standard errors. The sample in our integrative path model using FIML was 6,210, which represents the ALSPAC sample with data on general psychopathology at age 13.

## Results

### Step 1: General Psychopathology Factor

At age 13, all psychopathology sub-domains loaded significantly on the general psychopathology factor independent of the domain-specific internalizing and externalizing factors, with all but the specific phobia factor loading above 0.30 (see Table S3.1). Similarly, all internalizing sub-domains loaded significantly on the internalizing factor and all externalizing sub-domains loaded significantly on the externalizing factor independent of the general psychopathology factor, with all factor loadings above 0.30. The bifactor-specific model fit indices (see Table S3.4) supported a multidimensional conceptualization of general psychopathology, with the specific factors adding important variation to a unidimensional general factor. Specifically, the proportion of variance attributable to all sources of common variance was high (omega = 0.82), while the variance attributable to solely the single general factor was considerably lower (omega*H* = 0.48). This is supported by the moderate explained common variance of the general factor (ECV = 0.44), in conjunction with a modest percentage of uncontaminated correlations (PUC = 0.54). Construct replicability was borderline acceptable for the general factor (*H* = 0.69) using the 0.70 benchmark (Rodriguez et al. 2016b) and was slightly lower for the specific factors (*H*_internalizing_ = 0.65, *H*_externalizing_ = 0.57). However, it should be noted that construct replicability is highly influenced by the number of variables used to define these general (8 indicators) and specific internalizing (5 indicators) and externalizing (3 indicators) factors (Rodriguez et al. 2016a).

As displayed in Tables S3.1-S3.4, the bifactor structure of psychopathology was largely consistent across the three time points (7, 10, 13 years) in that (i) all psychopathology sub-domains loaded significantly on the general psychopathology factor and on one specific factor (internalizing, externalizing), and (ii) construct replicability was borderline acceptable for the general factor (range *H* = 0.64—0.70) and slightly lower for the specific internalizing (range *H* = 0.51 – 0.65) and externalizing (range *H* = 0.55—0.66) factors. The most notable difference is that the specific phobia sub-domain, which also had the lowest general factor loadings at ages 10 and 13, was excluded at age 7 due to non-convergence. Nevertheless, the general factor (*r* = 0.50–0.53, all *p* < 0.001) as well as the specific internalizing (*r* = 0.32–0.40, all *p* < 0.001) and externalizing (*r* = 0.42–0.56, all *p* < 0.001) factors were at least moderately correlated with themselves over time (i.e., significant autocorrelations).

### Step 2: The Association Between Exposure to Bullying and General Psychopathology

Correlations of bullying exposure with the general (*r*_age 7_ = 0.18, *r*_age 10_ = 0.15, *r*_age 13_ = 0.16, all *p* < 0.001) and the specific internalizing (*r*_age 7_ = 0.001, *r*_age 10_ = 0.02, *r*_age 13_ = 0.03, all *p* > 0.05) and externalizing (*r*_age 7_ = 0.17, *r*_age 10_ = 0.18, *r*_age 13_ = 0.12, all *p* < 0.001) factors defined in the bifactor model were similar across the three time points. Table 1 presents the linear regression results for bullying victimization in relation to general psychopathology and internalizing and externalizing factor scores from the bifactor model at age 13. We highlight here three findings. First, in the model correcting for sex and early-childhood environmental risk (model A), the continuous severity index of bullying exposure at age 8 and/or 10 significantly associated with general psychopathology (β = 0.12).

We then examined internalizing and externalizing factors, as defined in the bifactor model and the correlated two-factors model (CFI = 0.962, RMSEA = 0.055, SRMR = 0.047) in relation to bullying exposure. Although bullying exposure associated with both the internalizing (β = 0.08) and externalizing (β = 0.13) factors from the correlated two-factors model (see Table S4), only the externalizing (β = 0.10) and not the internalizing (β = 0.02) factor associated with bullying exposure in the bifactor model (see Table 1). Similarly, when partialling out the shared variance between the internalizing and externalizing factors from the correlated two-factors model, only the externalizing (β = 0.09) and not the internalizing (β = 0.01) factor remained associated with bullying exposure (see Table S4). As can be seen in Table S5, regression coefficients were similar in the imputed and non-imputed data sets (*N* = 5,125).

Second, as can be seen in Table 1, these associations of bullying exposure with the general (β = 0.07) and externalizing (β = 0.04) factors considerably attenuated but remained statistically significant after incorporating pre-existing psychopathology at age 7 in the regression models. However, regression coefficients remained largely unchanged when additionally correcting for IQ (see Table S6).

Finally, we highlight associations of the various characteristics of exposure to bullying measured in the study, including chronicity (unstable, stable) and type (overt, relational, combined). As shown in Table 1, the binary measure of any bullying experience at age 8 and/or 10 associated with both the general (β = 0.12) and the externalizing (β = 0.09) factors but not with the internalizing factor (β = 0.02). Regarding the chronicity of bullying victimization, both stable or unstable bullying victimization associated significantly with the general (β_stable_ = 0.12; β_unstable_ = 0.10) and externalizing (β_stable_ = 0.10; β_unstable_ = 0.07) factors. With regards to the different types of bullying exposures included in the study, particularly combined (both overt and relational) and overt only types associated with the general (β_combined_ = 0.08; β_overt_ = 0.09; β_relational_ = 0.04) and externalizing (β_combined_ = 0.06; β_overt_ = 0.07; β_relational_ = 0.03) factors.

### Step 3: Integrative Longitudinal Model of General Psychopathology

**Fig. 1:**
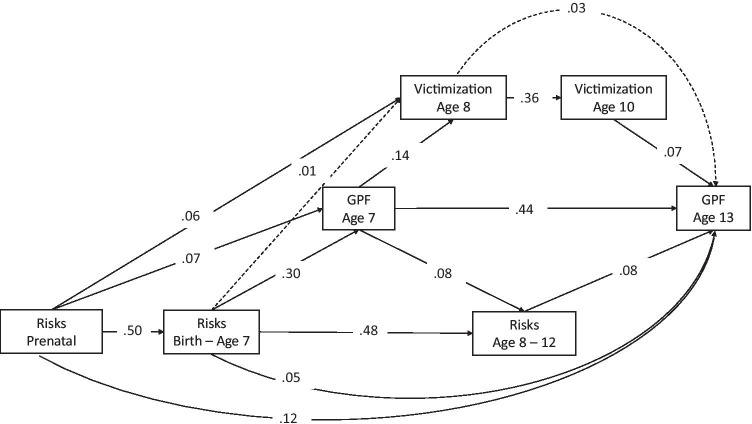
Prospective interrelations between cumulative risk exposure, bullying exposure, and the general psychopathology factor (GPF), *N* = 6,210. Solid arrowed lines indicate standardized path coefficients that survived bootstrap-corrected confidence intervals (i.e., significant paths)

As a final step, we made use of an integrative longitudinal model to examine the prospective interrelations between environmental risk exposure (prenatal, postnatal), the continuous severity index of exposure to bullying (age 8, 10) and general psychopathology (age 7, 13), while correcting for sex. Model fit was acceptable (RMSEA = 0.046, CFI = 0.988, SRMR = 0.027). Figure 1 shows that despite considerable continuity in the study variables (ranging from β = 0.36 to β = 0.50), higher levels of exposure to bullying at age 10 (β = 0.07) and both prenatal (β = 0.12) and postnatal (β = 0.05, β = 0.08) risk exposure independently associated with higher levels of general psychopathology at age 13. Higher general psychopathology levels at age 7 (β = 0.14) and prenatal risk exposure (β = 0.06) independently associated with higher levels of bullying victimization at age 8. Of note, associations were small in magnitude.

## Discussion

The primary aim of this study was to investigate prospective associations of exposure to bullying with general psychopathology factors across childhood, accounting for a wide range of pre- and postnatal factors. Using prospective data from a large birth cohort study, we found that exposure to bullying in primary school associated with the general psychopathology factor in early adolescence. Our integrative longitudinal model showed that pre-existing psychopathology, together with cumulative risk exposure, is also a vulnerability factor for bullying exposure.

Although it is well established that those whose are bullied are at increased risk for a wide range of mental health disorders and symptoms, less is known about the extent to which these findings are specific to these outcomes or reflect a general vulnerability to psychopathology. We found that bullying exposure was associated with both internalizing and externalizing factors from the correlated-factors model that has been commonly used in research on the structure of psychopathology. However, after extracting the shared variance that belongs to the general factor of psychopathology, effect estimates for the internalizing factor decreased considerably in size and dropped to insignificant. In the correlated two-factors model, the observed link between bullying exposure and the internalizing factor was likely inflated because the internalizing factor contained variance shared with the externalizing factor, which associated with bullying exposure. The bifactor model, however, has the advantage of directly assessing the shared variance between internalizing and externalizing domains (general factor), while simultaneously modeling the unique variance in each domain (specific factors).

Concerns have been raised, however, about the stability of these general and specific factors across samples or indicators (Bornovalova et al. 2020). Although we found support for a general factor of psychopathology with borderline acceptable construct replicability across three different time points (ages 7, 10, 13), the pattern of the factor loadings for the specific factors did not replicate well. This finding supports previous research by Gluschkoff et al. (2019), showing that despite an equal degree of strong longitudinal invariance for the correlated factors model and the bifactor model, the specific internalizing factor demonstrated unacceptable construct replicability estimates. For example, some of the factor loadings dropped to insignificant once the general factor was included in the model. Although these irregular loading patterns can challenge the interpretation of the specific factors, our longitudinal data demonstrated consistent, moderate autocorrelations over time, as well as consistent correlations with bullying exposure.

In particular, youth chronically exposed to multiple forms of victimization (i.e., both overt and relational) displayed greater levels of general psychopathology in early adolescence. This finding is in line with previous research indicating a dose–response relationship between chronic (8 and 10 years) or combined (overt and relational) exposure to bullying and borderline personality symptoms at age 11 years (Wolke et al. 2012). All of these associations considerably attenuated but remained significant after correcting for pre-existing general psychopathology. This finding supports prior evidence that the association between exposure to bullying and mental health outcomes can partially be accounted for by pre-existing vulnerabilities of bullied individuals (Hodges and Perry 1999; Reijntjes et al. 2010; Singham et al. 2017). Children exposed to bullying may differ from children not exposed in individual characteristics, such as having fewer friends, withdrawal or aggressiveness, which in turn increase their risks for being bullied (Arseneault et al. 2010; Monks et al. 2009). It has been previously shown that children who displayed aggressive behaviors in early childhood were more likely than nonaggressive children to experience chronic or high levels of bullying in preschool (Barker et al. 2008).

Integrating repeated assessments of general psychopathology (7, 13 years), exposure to bullying (8, 10 years) and environmental risk exposure (prenatal, early-childhood, late-childhood) in a longitudinal risk model, we also found that general psychopathology prospectively associated with bullying exposure, with higher levels of general psychopathology at age 7 rendering youth more susceptible to exposure to bullying at age 8. Bullying exposure at age 8, in turn, associated with general psychopathology at age 13 through its two-year continuity. Although statistically significant, the observed association of exposure to bullying with general psychopathology was small in magnitude when controlling for the various variables included in the model. This is in line with multifactorial influences on overall mental health problems (Lereya et al. 2015). For example, we found that the effect size of bullying exposure was similar to that observed for the cumulative risk exposure score, including a wide range of adversities known to be associated with mental health.

Self-reports involve children’s subjective perception of being victimized and likely tap into children’s feelings and well-being. Indeed, it has been demonstrated that self-reports of bullying victimization are stronger predictors of internalizing problems than peer reports (Bouman et al. 2012). However, this also implies that parents may have under-reported their children’s internalizing symptoms. Nevertheless, we found that the internalizing and externalizing factors were largely equally indicative of the general factor and were similarly associated with bullying exposure in the correlated-factors model. By using different reporters for bullying exposure and child outcomes, we were able to control for common method variance attributable to the informants.

The current findings should be interpreted in the context of several limitations. First, as mentioned previously, the replicability of the specific factors was sub optimal, owing in part to their small number of indicators. Therefore, in the future, our findings need to be replicated in longitudinal data that allows a higher number of psychiatric domains and disorders. Second, as with most longitudinal studies, considerable attrition occurred. Attrition might result in a loss of power to detect effects and may also bias the findings to those individuals who continued participating in the study. However, bullied children with missing data on covariates were as likely as children with complete data to have higher levels of psychopathology. This supports previous simulations using ALSPAC data, demonstrating that associations between predictors and outcomes are unlikely to be substantially altered by selective dropout (Wolke et al. 2009). Of note, the full information likelihood (FIML) approach enabled our integrative path model to be conducted on the full sample of children with available data on general psychopathology at age 13. Third, the analyses are correlational in nature and, hence, causality cannot be inferred. Fourth, in line with previous child studies, the current study examined the structure of psychopathology using the broadly defined internalizing and externalizing domains. Other disorders not studied here (e.g., obsessive–compulsive disorders, schizophrenia) might also contribute to a general factor of psychopathology (Caspi et al. 2014). Finally, although we controlled for a wide range of covariates, spanning the prenatal period up to early adolescence, the possibility of residual confounding cannot be fully excluded.

These limitations notwithstanding, this is the first population-based longitudinal study to show that exposure to bullying in primary school is a risk factor for a more general vulnerability to psychopathology in early adolescence. Although small in magnitude, the findings of the current study highlight the potential value of a transdiagnostic approach to understanding psychopathology.

## Electronic supplementary material

Below is the link to the electronic supplementary material.

Supplementary file1 (PDF 249 KB)

Supplementary file2 (XLSX 108 KB)
