# Supplementary material for: Exposure to Bullying and General Psychopathology: A Prospective, Longitudinal Study
Source: Res Child Adolesc Psychopathol. 2021 Jan 22;49(6):727–36. doi: 10.1007/s10802-020-00760-2 (PMC8096758; doi:10.1007/s10802-020-00760-2)
Supplement: Supplementary file 1 — Supplementary file1 (PDF 249 KB) [file 10802_2020_760_MOESM1_ESM.pdf]

S1.1 Environmental risk exposure items - Prenatal period

	Valid Range	*M (SD)*	*Scale*	*Time Points*	*Measurement Unit*	*Items*	
**Life events**	0-11	2.33 (1.72)	Life events scale	18w gestation	Dichotomous	Your partner died<br>One of your children died<br>A friend or relative died<br>One of your children was ill<br>Your partner was ill<br>A friend or relative was ill<br>You were admitted to hospital<br>You were very ill<br>Your partner lost his job<br>Your partner had problems at work<br>You had problems at work	You lost your job<br>You moved house<br>You were bleeding and thought you might miscarry<br>You started a new job<br>You had a test to see if your baby was abnormal<br>Result on a test that suggested your baby might not be normal<br>You were told that you were going to have twins<br>You heard something that happened might be harmful to the baby<br>You took an examination<br>Your house or car was burgled<br>You had an accident
**Contextual Risks**	0-5	0.55 (0.87)	Life events scale	18w gestation	Dichotomous	You had a major financial problem<br>You became homeless<br>Your income was reduced	
			Family adversity index	Pregnancy composite	Dichotomous	Housing adequacy<br>Housing Basic Living<br>Housing Defects<br>Financial difficulties	
**Parental Risks**	0-9	0.49 (0.79)	Life events scale	18w gestation	Dichotomous	You were in trouble with the law<br>Your partner was in trouble with the law<br>You were convicted of an offence<br>You attempted suicide<br>You tried to have an abortion	
			Family adversity index	Pregnancy composite	Dichotomous	Early parenthood<br>Maternal education<br>Psychopathology of mother<br>Substance abuse<br>Crime trouble with police<br>Crime convictions	
**Interpersonal Risks**	0-13	1.18 (1.51)	Life events scale	18w gestation	Dichotomous	You found that your partner didn't want your child<br>You argued with your partner<br>You had arguments with your family or friends<br>Your partner hurt you physically<br>Your partner hurt your children physically<br>Your partner was emotionally cruel to you	Your partner was emotionally cruel to your children<br>You were divorced<br>Your partner went away<br>You and your partner separated
			Family adversity index	Pregnancy composite	Dichotomous	Partner Status<br>Partner Affection<br>Partner cruelty<br>Family Size	Family Major problems<br>Partner Support<br>Social Network - Emotional<br>Social Network - Practical

Model fit indices for the prenatal period were $\chi2(2) = 163.581$, $p < .001$; CFI = .958; SRMR = 0.026 ; RMSEA = .075.

S1.2 Environmental risk items - Early Childhood (birth - age 7)

	Valid Range	*M (SD)*	*Scale*	*Time points*	*Measurement unit*	*Items*	
**Life Events**	0-83	27.51 (11.96)	Life events scale	8wk, 8m, 21m<br>3y, 4y, 5y, 6y	Dichotomous	Your partner died<br>One of your children died<br>A friend or relative died<br>One of your children was ill<br>Your partner was ill<br>A friend or relative was ill<br>You were admitted to hospital<br>You were very ill<br>Your partner lost his job<br>Your partner had problems at work<br>You had problems at work	You lost your job<br>You moved house<br>You were bleeding and thought you might miscarry<br>You started a new job<br>You had a test to see if your baby was abnormal<br>Result on a test that suggested your baby might not be normal<br>You were told that you were going to have twins<br>You heard something that happened might be harmful to the baby<br>You took an examination<br>Your house or car was burgled<br>You had an accident<br>You became pregnant<br>You returned to work<br>Your partner started a new job<br>Your pet died
			Life events - child	18m, 30m,<br>3y, 4y, 5y, 6y	Dichotomous	Child has been taken into care<br>Child's pet died<br>Child moved home<br>Child had shock or fright<br>Child has had someone in family die<br>Child has been separated from mother<br>Child has been separated from father	Child has had a new mother or father<br>Child has had a new brother or sister<br>Child has been admitted to hospital<br>Child has changed their caretaker<br>Child has been separated from someone else<br>Child has started a new school<br>Child has lost their best friend<br>Child started school
**Contextual Risks**	0-20	3.53 (3.05)	Life events scale	8wk, 8m, 21m<br>3y, 4y, 5y, 6y	Dichotomous	You had a major financial problem<br>You became homeless<br>Your income was reduced	
			Family adversity index	0-2y composite<br>2-4y composite	Dichotomous	Housing adequacy<br>Housing Basic Living<br>Housing Defects<br>Financial difficulties	
**Parental Risks**	0-23	1.21 (1.75)	Life events scale	8wk, 8m, 21m<br>3y, 4y, 5y, 6y	Dichotomous	You were in trouble with the law<br>Your partner was in trouble with the law<br>You were convicted of an offence<br>You attempted suicide<br>You tried to have an abortoin	
			Family adversity index	0-2y composite<br>2-4y composite	Dichotomous	Early parenthood<br>Maternal education<br>Psychopathology of mother<br>Substance abuse<br>Crime trouble with police<br>Crime convictions	
**Interpersonal Risks**	0-47	11.82 (7.52)	Life events scale	8wk, 8m, 21m<br>3y, 4y, 5y, 6y	Dichotomous	You found that your partner didn't want your child<br>You argued with your partner<br>You had arguments with your family or friends<br>Your partner hurt you physically	Your partner was emotionally cruel to you<br>You were divorced<br>Your partner went away<br>You and your partner separated
			Family adversity index	0-2y composite<br>2-4y composite	Dichotomous	Partner Status<br>Partner Affection<br>Partner cruelty<br>Family Size	Family major problems<br>Low partner support<br>Low social network, emotional<br>Low social network, practical
			Partner realtionship	21m, 33m, 6y	Nominal<br>*Collapsed*<br>*0 = No*<br>*1 = Yes, mom or/and partner*	Shouted in anger in past 3 months<br>Hit or slapped in past 3 months<br>Threw something in anger in past 3 mths	
			Harsh Parenting	18m, 2y, 3y<br>4y, 6y	Ordinal, coding system differed across time points<br>*Summed scores and performed median split*	Shout during tantrums/when naughty<br>Smack during tantrums/when naugthy	
**Direct victimization**	0-17	0.45 (1.24)	Life events - child	18m, 30m,<br>3y, 4y, 5y, 6y	Dichotomous	Child has been physically hurt by someone<br>Child has been sexually abused	
			Life events scale	8wk, 8m, 21m<br>3y, 4y, 5y, 6y	Dichotomous	Partner was physically cruel to child<br>Mother was physcially cruel to child<br>Partner was emotionally cruel to child<br>Mother was emotionally cruel to child	

$\chi2(5) = 708.866$, $p < .001$; CFI = .921; SRMR = 0.039; RMSEA = .099.

S1.3 Environmental risk items - Mid Childhood (age 8 - 12)

	Valid Range	*M (SD)*	*Scale*	*Time points*	*Measurement unit*	*Items*	
**Life Events**	0-30	7.27 (4.50)	Life events scale	9 y, 11 y	Dichotomous	Your partner died<br>One of your children died<br>A friend or relative died<br>One of your children was ill<br>Your partner was ill<br>A friend or relative was ill<br>You were admitted to hospital<br>You were very ill<br>Your partner lost his job<br>Your partner had problems at work<br>You had problems at work	You lost your job<br>You moved house<br>You were bleeding and thought you might miscarry<br>You started a new job<br>You had a test to see if your baby was abnormal<br>Result on a test that suggested your baby might not be normal<br>You were told that you were going to have twins<br>You heard something that happened might be harmful to the baby<br>You took an examination<br>Your house or car was burgled<br>You had an accident<br>You returned to work<br>One of your children started school<br>Your partner started a new job<br>Your pet died
			Life events - child	9y	Dichotomous	Child has been taken into care<br>Child's pet died<br>Child moved home<br>Child had shock or fright<br>Child has had someone in family die<br>Child has been separated from mother<br>Child has been separated from father	Child has had a new mother or father<br>Child has had a new brother or sister<br>Child has been admitted to hospital<br>Child has changed their caretaker<br>Child has been separated from someone else<br>Child has started a new school<br>Child has lost their best friend
**Contextual Risks**	0-6	0.50 (0.82)	Life events scale	9y , 11y	Dichotomous	You had a major financial problem<br>You became homeless<br>Your income was reduced	
**Parental Risks**	0-4	0.06 (0.29)	Life events scale	9y, 11y	Dichotomous	You were in trouble with the law<br>Your partner was in trouble with the law<br>You were convicted of an offence<br>You attempted suicide<br>You tried to have an abortoin	
**Interpersonal Risks**	0-15	2.05 (2.09)	Life events scale	9y, 11y	Dichotomous	You found that your partner didn't want your child<br>You argued with your partner<br>You had arguments with your family or friends<br>Your partner hurt you physically	Your partner was emotionally cruel to you<br>You were divorced<br>Your partner went away<br>You and your partner separated
			Partner realtionship	9y	Nominal<br>*Collapsed*<br>*0 = No*<br>*1 = Yes, mom or/and partner*	Shouted in anger in past 3 months<br>Hit or slapped in past 3 months<br>Threw something in anger in past 3 mths	
			Harsh Parenting	9y	Ordinal,<br>*Collapsed*<br>*0 = Never*<br>*1 = Rarely - every day*	Child slapped/hit	
**Direct victimization**	0-7	0.10 (0.41)	Life events - child	9y	Dichotomous	Child has been physically hurt by someone<br>Child has been sexually abused	
			Life events scale	9y, 11y	Dichotomous	Partner was physically cruel to child<br>Mother was physcially cruel to child<br>Partner was emotionally cruel to child<br>Mother was emotionally cruel to child	

Model fit indices for the age 8 – 12 period were $\chi2(5) = 132.147$, $p < .001$; CFI = .947; SRMR = 0.029 ; RMSEA = .051.

Table S2 Correlations among study variables

		Severity index (8, 10y), score	Any exposure (8, 10y), yes vs no	General psychopathology factor (13y)	Internalizing factor (13y)	Internalizing factor (13y), correlated factors model	Externalizing factor (13y)	Externalizing factor (13y), correlated factors model	Sex. girl vs boy	Prenatal risk	Early-childhood risk (0-7y)	Late-childhood risk (8-12y)	IQ
Severity index (8, 10y), score	Pearson Correlation	1	.644**	.159**	0.026	.096**	.123**	.167**	-.065**	.100**	.116**	.080**	-.092**
	Sig. (2-tailed)		0.000	0.000	0.058	0.000	0.000	0.000	0.000	0.000	0.000	0.000	0.000
	N	5370	4613	5370	5370	5370	5370	5370	5370	5125	5125	5295	4650
Any exposure (8, 10y), yes vs no	Pearson Correlation	.644**	1	.148**	0.018	.084**	.116**	.159**	-.081**	.102**	.097**	.053**	-.079**
	Sig. (2-tailed)	0.000		0.000	0.231	0.000	0.000	0.000	0.000	0.000	0.000	0.000	0.000
	N	4613	4613	4613	4613	4613	4613	4613	4613	4423	4423	4559	4257
General psychopathology factor (13y)	Pearson Correlation	.159**	.148**	1	.349**	.783**	.557**	.911**	-0.020	.266**	.308**	.247**	-.192**
	Sig. (2-tailed)	0.000	0.000		0.000	0.000	0.000	0.000	0.124	0.000	0.000	0.000	0.000
	N	5370	4613	6210	6210	6210	6210	6210	6210	5953	5953	6029	4675
Internalizing factor (13y)	Pearson Correlation	0.026	0.018	.349**	1	.844**	-.212**	.124**	.197**	.095**	.142**	.128**	0.006
	Sig. (2-tailed)	0.058	0.231	0.000		0.000	0.000	0.000	0.000	0.000	0.000	0.000	0.672
	N	5370	4613	6210	6210	6210	6210	6210	6210	5953	5953	6029	4675
Internalizing factor (13y), correlated factors model	Pearson Correlation	.096**	.084**	.783**	.844**	1	.148**	.564**	.133**	.210**	.263**	.222**	-.100**
	Sig. (2-tailed)	0.000	0.000	0.000	0.000		0.000	0.000	0.000	0.000	0.000	0.000	0.000
	N	5370	4613	6210	6210	6210	6210	6210	6210	5953	5953	6029	4675
Externalizing factor (13y)	Pearson Correlation	.123**	.116**	.557**	-.212**	.148**	1	.835**	-.103**	.154**	.178**	.134**	-.163**
	Sig. (2-tailed)	0.000	0.000	0.000	0.000	0.000		0.000	0.000	0.000	0.000	0.000	0.000
	N	5370	4613	6210	6210	6210	6210	6210	6210	5953	5953	6029	4675
Externalizing factor (13y), correlated factors model	Pearson Correlation	.167**	.159**	.911**	.124**	.564**	.835**	1	-.071**	.246**	.285**	.222**	-.206**
	Sig. (2-tailed)	0.000	0.000	0.000	0.000	0.000	0.000		0.000	0.000	0.000	0.000	0.000
	N	5370	4613	6210	6210	6210	6210	6210	6210	5953	5953	6029	4675
Sex	Pearson Correlation	-.065**	-.081**	-0.020	.197**	.133**	-.103**	-.071**	1	0.008	-0.007	-0.007	-0.013
	Sig. (2-tailed)	0.000	0.000	0.124	0.000	0.000	0.000	0.000		0.538	0.578	0.574	0.388
	N	5370	4613	6210	6210	6210	6210	6210	6210	5953	5953	6029	4675
Prenatal risk	Pearson Correlation	.100**	.102**	.266**	.095**	.210**	.154**	.246**	0.008	1	.497**	.257**	-.128**
	Sig. (2-tailed)	0.000	0.000	0.000	0.000	0.000	0.000	0.000	0.538		0.000	0.000	0.000
	N	5125	4423	5953	5953	5953	5953	5953	5953	5953	5953	5790	4675
Early-childhood risk (0-7y)	Pearson Correlation	.116**	.097**	.308**	.142**	.263**	.178**	.285**	-0.007	.497**	1	.507**	-.042**
	Sig. (2-tailed)	0.000	0.000	0.000	0.000	0.000	0.000	0.000	0.578	0.000		0.000	0.004
	N	5125	4423	5953	5953	5953	5953	5953	5953	5953	5953	5790	4675
Late-childhood risk (8-12y)	Pearson Correlation	.080**	.053**	.247**	.128**	.222**	.134**	.222**	-0.007	.257**	.507**	1	-0.014
	Sig. (2-tailed)	0.000	0.000	0.000	0.000	0.000	0.000	0.000	0.574	0.000	0.000		0.355
	N	5295	4559	6029	6029	6029	6029	6029	6029	5790	5790	6029	4632
IQ	Pearson Correlation	-.092**	-.079**	-.192**	0.006	-.100**	-.163**	-.206**	-0.013	-.128**	-.042**	-0.014	1
	Sig. (2-tailed)	0.000	0.000	0.000	0.672	0.000	0.000	0.000	0.388	0.000	0.004	0.355	
	N	4650	4257	4675	4675	4675	4675	4675	4675	4675	4675	4632	4675
Mean (SD) or %		4.58 (4.94)	53	0.05 (0.64)	0.01 (0.65)	0.04 (0.74)	0.04 (0.60)	0.06 (0.76)	51	-0.07 (0.74)	0.32 (0.83)	0.06 (0.82)	105.61 (16.34)

**. Correlation is significant at the 0.01 level (2-tailed).

Figure S1 Three different models of psychopathology

Model A: Correlated factors

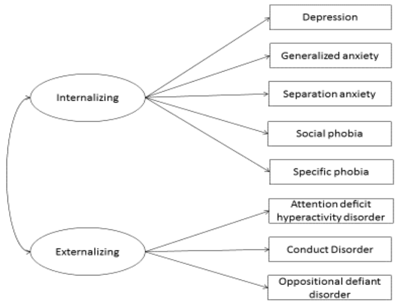

Model B: Bifactor

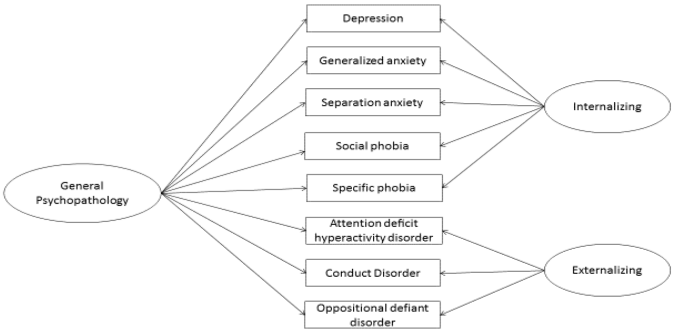

Model C: One factor

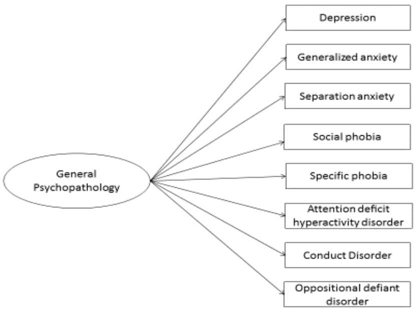

Table S3.1 The structure of psychpathology at age 13 (N = 6,210): Model fit statistics, loadings, and correlations of thee different models

	Correlated factors model (A)			Bifactor model (B)				1-factor model (C)	
Statstics, loadings, and correlations	Model fit	Externalizing	Internalizing	Model fit	General	Externalizing	Internalizing	Model fit	General
Statistics									
Comparative fit index	0.962			0.992				0.764	
Root-mean-square error of approximation	0.055			0.032				0.136	
Standardized Root Mean Square Residual	0.047			0.020				0.116	
Standardized factor loadings									
Attention deficit hyperactivity disorder		0.785			0.656	0.388			0.732
Conduct disorder		0.676			0.486	0.446			0.607
Oppositional defiant disorder		0.778			0.518	0.686			0.685
Major depresive disorder			0.658		0.517		0.350		0.541
Generalized anxiety disorder			0.665		0.318		0.698		0.471
Separation anxiety			0.690		0.427		0.514		0.564
Social phobia			0.521		0.322		0.391		0.42
Specific phobia			0.376		0.110		0.446		0.259
Factor correlation			0.437						

Table S3.2 The structure of psychpathology at age 7 (N = 7,814): Model fit statistics, loadings, and correlations of thee different models

	Correlated factors model (A)			Bifactor model (B)				1-factor model (C)	
Statstics, loadings, and correlations	Model fit	Externalizing	Internalizing	Model fit	General	Externalizing	Internalizing	Model fit	General
Statistics									
Comparative fit index	0.985			0.998				0.793	
Root-mean-square error of approximation	0.038			0.019				0.139	
Standardized Root Mean Square Residual	0.031			0.012				0.109	
Standardized factor loadings									
Attention deficit hyperactivity disorder		0.781			0.589	0.466			0.731
Conduct disorder		0.588			0.420	0.396			0.537
Oppositional defiant disorder		0.753			0.477	0.663			0.682
Major depresive disorder			0.664		0.486		0.383		0.521
Generalized anxiety disorder			0.673		0.337		0.743		0.464
Separation anxiety			0.620		0.434		0.396		0.484
Social phobia			0.475		0.271		0.385		0.353
Factor correlation			0.432						

Table S3.3 The structure of psychpathology at age 10 (N = 7,145): Model fit statistics, loadings, and correlations of thee different models

	Correlated factors model (A)			Bifactor model (B)				1-factor model (C)	
Statstics, loadings, and correlations	Model fit	Externalizing	Internalizing	Model fit	General	Externalizing	Internalizing	Model fit	General
Statistics									
Comparative fit index	0.976			0.993				0.793	
Root-mean-square error of approximation	0.044			0.030				0.127	
Standardized Root Mean Square Residual	0.039			0.019				0.104	
Standardized factor loadings									
Attention deficit hyperactivity disorder		0.781			0.465	0.583			0.721
Conduct disorder		0.626			0.343	0.516			0.564
Oppositional defiant disorder		0.778			0.396	0.725			0.689
Major depresive disorder			0.609		0.622		0.127		0.514
Generalized anxiety disorder			0.699		0.569		0.395		0.519
Separation anxiety			0.663		0.520		0.420		0.545
Social phobia			0.457		0.434		0.146		0.386
Specific phobia			0.426		0.211		0.609		0.316
Factor correlation			0.459						

Table S3.4 Bifactor-specific model fit indices

General psychopathology, age 13

	g	EXT	INT	Unique
Total variance	,200	,103	,153	,544
Common Var. (ECV)	,439	,225	,336	
Omega	,816	,803	,735	
Omega(h)	,475	,366	,491	
Relative Omega	,582	,456	,667	
Factor correlation	,689	,605	,700	
H	,693	,568	,652	
PUC	,536			

General psychopathology, age 7

	g	EXT	INT	Unique
Total variance	.195	.116	.143	.546
Common Var. (ECV)	.428	.256	.316	
Omega	.798	.760	.715	
Omega(h)	.481	.390	.435	
Relative Omega	.604	.513	.609	
Factor correlation	.694	.624	.660	
H	.641	.555	.638	
PUC	.571			

General psychopathology, age 10

	g	EXT	INT	Unique
Total variance	,213	,141	,093	,553
Common Var. (ECV)	,477	,316	,207	
Omega	,810	,776	,735	
Omega(h)	,544	,541	,251	
Relative Omega	,671	,697	,342	
Factor correlation	,737	,735	,501	
H	,702	,665	,507	
PUC	,536			

Table S4. Associations between exposure to bullying and interalizing or externalizing factors in a correlated factors model

	Model A		Model B		Model C	
	INT	EXT	INT	EXT	INT	EXT
Bullying exposure	β (SE)	β (SE)	β (SE)	β (SE)	β (SE)	β (SE)
Severity index (*n* = 5,370)[a], score	0.08 (0.01)***	0.13 (0.01)***	0.06 (0.01)***	0.06 (0.01)***	0.01 (0.01)	0.09 (0.01)***
Any exposure (*n* = 4,613), yes vs no	0.07 (0.01)***	0.13 (0.01)***	0.05 (0.01)***	0.06 (0.01)***	0.00 (0.01)	0.09 (0.01)***
Chronicity (*n* = 4,613)						
none (reference)						
unstable	0.06 (0.02)***	0.10 (0.02)***	0.04 (0.01)**	0.04 (0.01)**	0.01 (0.01)	0.07 (0.01)***
stable	0.06 (0.02)***	0.13 (0.02)***	0.04 (0.02)**	0.07 (0.02)***	-0.01 (0.01)	0.10 (0.01)***
Type (*n* = 4,882)[b]						
none (reference)						
overt only	0.05 (0.01)**	0.10 (0.02)***	0.03 (0.01)**	0.05 (0.01)***	-0.01 (0.01)	0.07 (0.01)***
relational only	0.03 (0.02)*	0.04 (0.01)**	0.02 (0.01)	0.03 (0.01)*	0.01 (0.01)	0.02 (0.01)
both	0.05 (0.01)***	0.09 (0.02)**	0.04 (0.01)**	0.05 (0.01)***	0.00 (0.01)	0.06 (0.01)***

Note: Model A presents linear regression results controlling for sex and early-life environmental risk exposure; Model B is similar to model A, additionally controlling for pre-existing psychopathology (GP, INT, or EXT); Model C is similar to model A, additionally controlling the internalizing factor for the externalizing factor and vice versa.

[a] Summed across time points (age 8 and 10 years).

[b] Measured at age 10

*p<0.05; **p <0.01; ***p<0.001

INT = internalizing factor; EXT = externalizing factor

Table S5. Associations between exposure to bullying and interalizing or externalizing factors in non-imputed data

	Model A: bifactor model			Model A: correlated factors model	
	GPF	INT	EXT	INT	EXT
Bullying exposure	β (SE)	β (SE)	β (SE)	β (SE)	β (SE)
Severity index (*n* = 5,125)[a], score	0.13 (0.01)***	0.02 (0.01)	0.10 (0.01)***	0.07 (0.01)***	0.013 (0.01)***

Note: Model A presents linear regression results controlling for sex and early-life environmental risk exposure.

[a] Summed across time points (age 8 and 10 years).

*p<0.05; **p <0.01; ***p<0.001

INT = internalizing factor; EXT = externalizing factor

Table S6. Associations between exposure to bullying and general or specific factors of psychopathology

	Minimally adjusted model			Fully adjusted model		
	GP	INT	EXT	GP	INT	EXT
Bullying exposure	β (SE)	β (SE)	β (SE)	β (SE)	β (SE)	β (SE)
Severity index (n = 5,370)a, score	0.16 (0.01)***	0.04 (0.01)**	0.12 (0.01)***	0.06 (0.01)***	0.03 (0.01)*	0.04 (0.01)**
Any exposure (n = 4,613), yes vs no	0.15 (0.01)***	0.03 (0.01)*	0.11 (0.01)***	0.05 (0.01)***	0.02 (0.01)	0.04 (0.01)**
Chronicity (n = 4,613)						
none (reference)						
unstable	0.12 (0.02)***	0.04 (0.02)*	0.09 (0.02)***	0.04 (0.01)**	0.03 (0.01)*	0.02 (0.01)
stable	0.15 (0.02)***	0.01 (0.02)	0.12 (0.02)***	0.06 (0.01)***	0.00 (0.02)	0.05 (0.02)***
Type (n = 4,882)b						
none (reference)						
overt only	0.11 (0.02)***	0.01 (0.01)	0.08 (0.02)***	0.05 (0.01)***	0.00 (0.01)	0.04 (0.01)*
relational only	0.04 (0.02)**	0.02 (0.02)	0.03 (0.02)	0.02 (0.01)	0.01 (0.02)	0.02 (0.01)
both	0.10 (0.02)***	0.02 (0.01)	0.07 (0.02)***	0.05 (0.01)***	0.02 (0.01)	0.03 (0.01)*
Status (n = 4,909)b						
none (reference)						
pure victim	0.11 (0.02)***	0.02 (0.01)	0.07 (0.01)***	0.06 (0.01)***	0.02 (0.01)	0.03 (0.01)*
pure bully	0.04 (0.01)**	0.02 (0.01)	0.02 (0.02)	0.03 (0.01)*	0.02 (0.01)	0.01 (0.02)
victim/bully	0.12 (0.02)***	0.02 (0.02)	0.10 (0.02)***	0.05 (0.01)***	0.01 (0.02)	0.05 (0.02)**

Note: Model A presents linear regression results controlling for sex; Model B is similar to model A, additionally controlling for early-life environmental risk exposure, pre-existing psychopathology (GP, INT, or EXT), and IQ.

a Summed across time points (age 8 and 10 years).

b Measured at age 10

*p<0.05; **p <0.01; ***p<0.001

GP= general psychopathology factor; INT = specific internalizing factor; EXT = specific externalizing factor
